# FinTech’s rapid growth and its effect on the banking sector

**DOI:** 10.1007/s42786-022-00045-w

**Published:** 2022-11-28

**Authors:** Charalampos Basdekis, Apostolos Christopoulos, Ioannis Katsampoxakis, Aikaterini Vlachou

**Affiliations:** 1 Department of Economics, National & Kapodistrian University of Athens, Athens, Greece; 2 Hellenic Open University, Patra, Greece; 3 University of West Attica, Egaleo, Greece; 4 Department of Business Administration, University of the Aegean, Chios, Greece; 5 Department of Statistics and Actuarial, Financial Mathematics, University of the Aegean, Samos, Greece

**Keywords:** FinTech, Big Tech, RegTech, Blockchain, APIs, Banking Industry, Regulation, Covid-19 pandemic

## Abstract

FinTech is a New Financial Technology, which provides financial services through innovative information and communication technologies. It is widely accepted that 4th industrial revolution, has affected tremendously the living and working conditions of the societies. The convergence between advanced technologies, entrepreneurship becomes more complex and remarkably computerized. Within such significant changes it is rather expected that banking, has been one of the most challenged sectors. New players like FinTech and Big Tech companies try to capitalize the circumstances, by promoting new consumer patterns to gain market shares. The purpose of this study is to investigate the rapid expansion of FinTech and to evaluate its impact on the Greek banking system. This topic becomes very important nowadays as the number of FinTech companies, which compete with traditional banks on financial products and services, are increasing constantly as digital technology develops. In our study we apply a questionnaire method mainly with closed questions to collect data from the main players. To do this, we use two questionnaires each one for a different sample. The first sample consists of the consumers for financial products and services in the Greek banking sector and the second sample consists of the employees in the Greek banking sector. According to the results, customers of all ages seem to trust the traditional banks more than FinTech companies whereas the level of mobile transactions separately for each consumer, depends on age and education. From the answers of the consumers, it is clear that security is on the top of their worries for using financial services by FinTech companies. On the other hand, the second questionnaire with bank employees shows clearly that educational level is a critical factor for their readiness and response to new technologies.

**Research Problem**: Advances in financial technology has brought a new era in the banking sector, introducing challenges, opportunities and risks. The integration of FinTech in the financial system has created global digital technology platforms through which new innovative financial products and services are provided to end customers.

At the same time, traditional banks seem unable to assimilate at the same level these new technologies to deliver products and services to their own customers. In the light of these developments, the question arises as to whether non-banking entities, such as FinTech companies, are capable to lead the competition in such a way and at such a level that banking products and services will no longer be a privilege of the traditional banking sector.

**Objective:** The evaluation of FinTech growth and the examination of the prospects for the Greek commercial banks, taking into consideration the presence of FinTech companies.

**Research Questions**:

1. What types of products and services are provided by FinTech companies and which of them are most attractive to users?
2. Which are the most important factors influencing the choice of FinTech services?
3. At what level traditional banks operating in Greece are interested to invest in FinTech?
4. How FinTech affects employment in the Greek banking sector?

## Introduction

In recent years, technology has developed rapidly, affecting the way and means in which financial products and services are provided and the way in which consumers are served. Looking in the past, we find Bill Gates, the founder of Microsoft to declare in 1990 that "banking is necessary, banks are not." Thirty years later, this statement seems to be more suitable than ever, especially in Europe [19]. One can surely say that banks in the future will have many different forms. Brei et al. [13] point out that in order to maintain their profitability in such an environment, many banks have placed great emphasis on fee-generating services.

It is noteworthy that in the age of advanced technological developments, even the nature of deposits is changing. This prompts Braggion et al. [12] to investigate whether FinTech expansion could pose a threat for financial stability. Already today banks accept deposits and make transactions in a digital form. However, at the same time, this raises a number of issues, such as resilience, security and competition in payments, the way financial services are provided, the way and security of cross-border money transfers, but also raises the question of private and public money issuance.

According to Arner et al. [3], the global financial crisis in 2008 proved to be the most critical moment for the strengthening of FinTech financial technology and RegTech regulatory technology, as it stimulated all processes much faster. Indeed, as banks are unable to adopt right away new technologies due to regulatory restrictions [25] they have to rely at least for some time on obsolete infrastructure technologies. Therefore, advances in technologies are expected to benefit the FinTech companies more. However, according to Philippon [45], this advantage for FinTech companies does not show reduction in the intermediation costs of the banking sector. At a European level, during the crisis of 2008, the primary concern of ECB was to introduce effective measures, in order to achieve the key objective of financial stability in Eurozone [32] and thereafter to move towards the introduction and development of a strict and effective regulation framework for the operation of FinTech companies.

The growth of FinTech companies has strengthened more, after the global financial crisis of 2007. Estimates by Finances Online indicate that there are currently more than 12,000 FinTech companies operating worldwide [33]. The main target of FinTech companies is to offer in a friendly way financial products and services to their customers, in a more efficient, transparent and more automated way [21]. In another recent study, Broby [14] concluded that, in an increasingly digital world, trust will remain at the core of banking, which means that transformation of assets will continue to play an important role. However, the nature of banking and financial services is expected to change dramatically. The technological achievements and the importance on R&D expenditures is of paramount importance for every business in or out of the financial sector [10, 30, 31].

Mitra and Karathanasopoulos [41] examined the impact of financial technology on the relative value of the business in the banking sector. They found that financial technologies affect operational risk and thus companies must take into account the benefits but also the risks from implementing new technological innovations.

Before the introduction of FinTech, entrepreneurs and individuals had to visit a bank branch to apply for small business credit lines, finance leases, mortgages, business loans, credit cards and various other banking services. However, after the introduction of FinTech companies people no longer need to visit a bank to apply for a mortgage loan or a consumer loan. The applications for these products are now offered online through FinTech companies [29] which are incorporated in various business models.

There is a wide variety of business models that have been established under the banner of Fintech such as, crowdfunding, payments, wealth management, lending, capital markets and insurance services. Every business model is unique but depends on the digital platform in order to reduce operating costs [26].

The findings of Karsh and Abuhara [29] show that FinTech companies will grow faster in an environment, where digital technology is available and the penetration of smartphones is high. The empirical results of this study show that the profitability of traditional banks is higher when they collaborate with FinTech companies and when the banks adopt their own financial technology in their business model.

Internationally, the most influential banking system by FinTech is in China. Arner et al. [6], report that although the structure of the Chinese banking system is inefficient, the penetration of technology is high and thus why we see in China a rapid increase of technology companies like Alibaba, Baidu and Tencent which have a significant impact on financial services [36]. Although, for at least one decade, those involved with FinTech have attracted the worldwide attention, this issue has not studied widely by academics.

The existing literature shows that although FinTech companies perform like banks, till today they are not regulated like banks.

The main goal of our study is to enlighten aspects of the rapid growth of the financial industry in combination with high technology. At the same time, we aim to clarify the role of FinTech in the financial sector in general, emphasizing though in the banking sector. The results of our study show that generation Z will be the basis for new era emerging in the banking system and their needs and expectations will act a key role. At the same time another important output is the interrelation between FinTech and prudent investments decisions.

The present work is structured as follows: The second section presents the historical development of FinTech and TechFin, the third section deals with the regulation framework of RegTech in Europe and the fourth section refers to the technology used and efficiency of FinTech. In the fifth section, we analyze the technological trends in the banking sector, and in the sixth one the impact of Covid-19 pandemic on FinTech. The methodological framework, the results and the empirical analysis are presented form the seventh to ninth sections while in the last section there are analyzed the conclusions and the discussion regarding the existing literature and the empirical research, giving answers to the research questions that we have identified, suggesting future research perspectives.

## The historical development of FinTech and TechFin

The term “FinTech” refers to companies that combine the provision of financial services with modern and innovative technologies, although the traditional banking sector has the potential for technological improvement and banks are working in this direction. However, in addition to the banking sector, there are FinTech companies that also offer insurance and financial instruments, either directly or as third parties. FinTech therefore includes companies that provide advance technology to financial service providers. However, it should be noted that there is a huge variation in the legislative and regulatory obligations that apply between banking institutions and FinTech companies [21].

According to the Financial Stability Board (FSB) [23], “FinTech is a new financial industry that applies new technology to improve financial activities, including processes, products or even business models”.

The development of FinTech can be divided into three main time periods [5]: The first is defined from 1866 to 1967 and focuses on the development of the infrastructure of economic globalization. The second refers to the period from 1967 to the outbreak of the international financial crisis and is characterized by the transition to digital technology. A hallmark of this period is the emergence of ATMs (Automated Teller Machine), the foundation of NASDAQ as the world's first digital stock exchange and the World Bank Interbank Financial Telecommunications Company (SWIFT), which is a network of encrypted messages that transmit secure information and instructions. [5].

FinTech’s most recent achievements during this period are the development of e-banking and e-commerce which resulted in a huge impact of the banking system on everyday human life. [5].

**Fig. 1:**
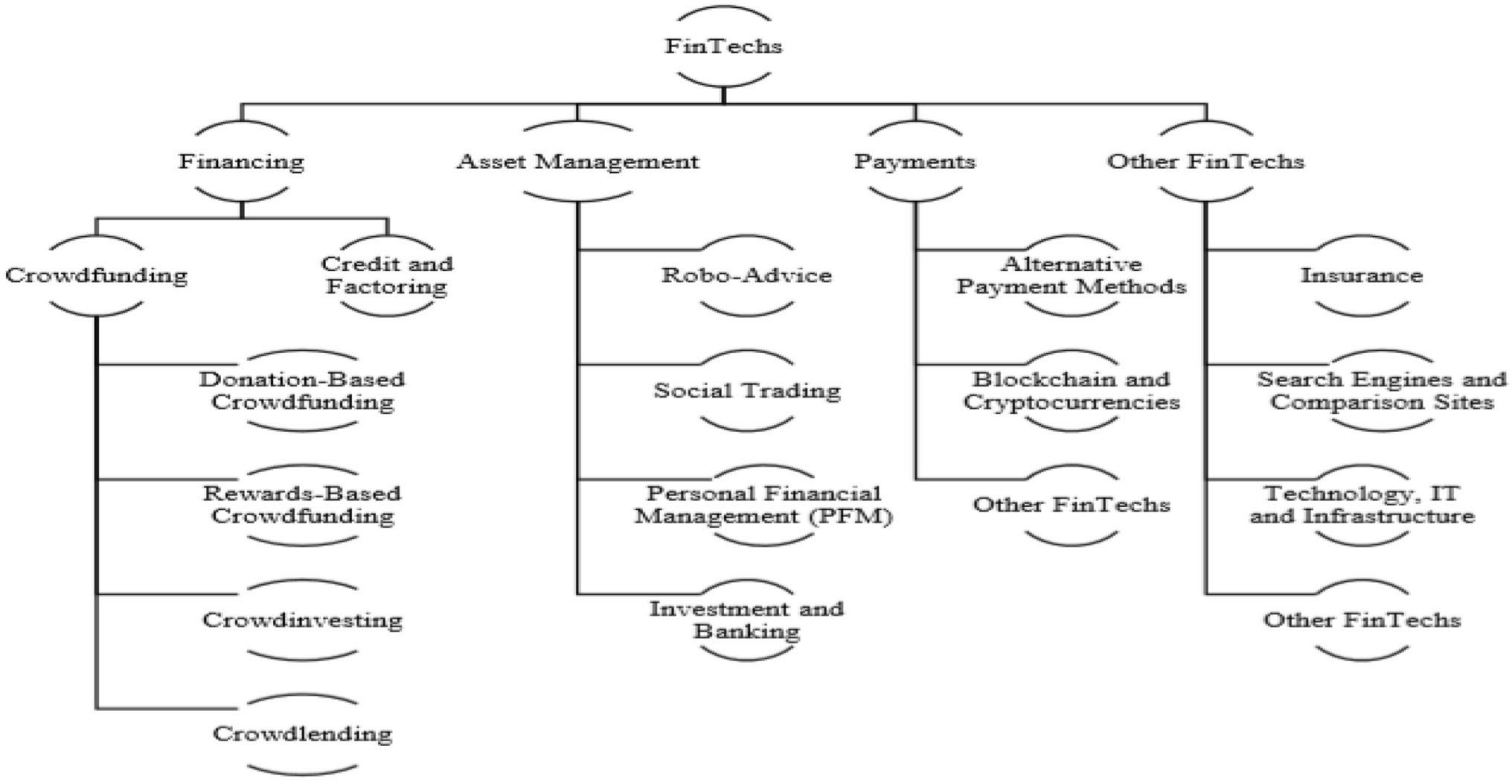
Categorization of Fintech firms Source: Dorfleitner et al. [21]

Currently we face the third era of FinTech, which corresponds to the response of distrust towards the performance of the traditional banking system. This period is characterized by the introduction of cryptocurrencies and the widespread use of smart phones, which allow the execution of several financial services. In fact, in the last decade, Google's digital wallet and Apple Pay have been introduced, which allow their holders to make electronic payments. Arner et al. [4], consider that in contrast with the more developed economies, in the emerging economies of Asia and Africa, FinTech has begun to develop in recent years. Dorfleitner et al. [21], point out that FinTech companies can be divided into four main categories, depending on the sector they operate: financing sector, asset management sector, payment transactions and other FinTech (see Fig. 1).

In addition to FinTech which act as financial intermediaries there are also data intermediaries TechFin companies which aim to take advantage of their relationship with customers in non-financial services to collect big data in order to provide them with purely financial products and services. TechFin companies create large-scale databases which allows them to offer financial services and become major non-banking players in the sector [57]. At the same time, FinTech companies may fill the gap or malfunction of the traditional banking system due to regulatory changes and the lack of technological and digital focus on the customer, providing solutions either directly or through the provision of know-how to existing banking providers.

## The regulatory regime of FinTech firms in Europe

The global financial crisis in 2007 significantly affected financial services and had a catalytic effect on FinTech growth. Although several years have passed it was only recently that legislation and the implementation of some international regulations became mandatory for these companies. Regulatory provisions primarily appeared with the introduction of Regulatory Technology (Reg Tech) which expanded rapidly as a result of the growth of FinTech companies. In turn, the growth of FinTech companies, has attracted the interest of the banking industry, regulators and consumers. The aim of RegTech companies is to provide secure, cost-effective and reliable regulatory solutions through the latest digital technology [20].

After the crisis, there was an economic gap, mainly due to the loss of confidence in traditional banking institutions. New Regulations through Basel III resulted in increased costs for these institutions due to the new regulatory obligations they had to comply with and their obligation to perform stress tests on a frequent basis.

All these developments have led in the growth of FinTech industry, which competes credit institutions by providing cheap and innovative services. In the field of payment services, the original PSD (Payments Services Directive—PSD) (Directive 2007/64/EU) was strengthened the competition in the European Market and the Simple European Payment Area (SEPA). The PSD2 Directive that followed not only helped to broaden the definition of payment services, but also extended the categories of providers [42]. Both directives define and extend the information, requirements, rights and obligations of users, as well as payment services that facilitate money transfers [56]. However, the year 2018 can be said with certainty to be the year that changed the game for traditional banks and this is mainly due to the revised payment directive PSD2.

The main objectives of the PSD2 Directive [19] are to:

- Contribute to the completion of an efficient European payment market.
- Contribute to improving fair competition between banking and non-banking providers regarding payment services.
- Promote competition in the new economic environment, where new innovative products and services are available.
- Offer secure payments.
- Reduce customer costs.

According to Navaretti et al. [42], almost all central banks in the EU have created innovation hubs that provide regulatory sandbox. In Greece, the FinTech Hub, which first appeared in March 2019, aims to enhance the interaction between banks and to facilitate the interaction of FinTech companies with the supervisory mechanism, which aims to enter the industry. Through this hub, it becomes clear that economic innovation is encouraged and implemented and therefore, in some way, a balance between risks and opportunities is ensured. This security has provided some form of support to businesses and individuals who are developing or considering introducing innovative products and services.

Due to the trend of convergence between banks and FinTech, the regulations focus mainly on the services provided and not on the provider and the main goal is to ensure that services and products are offered in full transparency [42].

To date, it seems that the financial services sector is undergoing a period of radical transformation. As a result, market forces and regulations are leading to the rapid growth of Open Banking. The legal basis is provided by the implementation of the PSD2 Directive creating a single Pan-European payment market. Third party access to customer data held by banks is also regulated and consequently banks cease to have exclusivity in their customer data [17].

## Technology and efficiency of FinTech

Numerous analysts argue that although FinTech’s original goal was to eradicate traditional banks from the market by acquiring a dominant position, there are several cases where we can see partnerships between these companies and established traditional banking institutions. In this way, the FinTech companies were able to cope with the difficulties they had in increasing the number of their customers by achieving larger economies of scale.

According to Vives [54], the use of new technologies has significant implications for the financial sector, such as reducing transaction costs and the availability of new and higher quality products.

One could briefly claim that:

- Big Data and the appropriate statistical models can control prospective borrowers more effectively, which is very important for tackling the problem of asymmetric information.
- Allows targeted pricing policies as sophisticated interest rate models are used.
- Less developed countries have access to financial services but also companies that until now did not have easy access to the banking system.
- Through new technologies, the business plan can be implemented and served more efficiently.

## Technological trends in banking sector

**Fig. 2:**
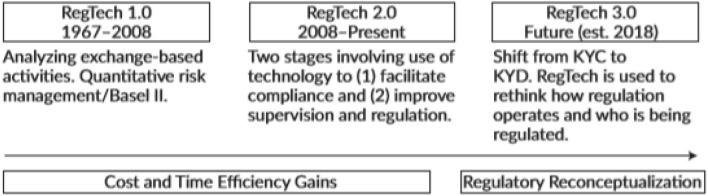
Stages of RegTech Development. Source: Arner et al. [2]

The growth of FinTech through newly established FinTech companies, contributed to the development of RegTech, due to the increased exposure to risk and the need for regulatory compliance. RegTech is considered as an evolving subcategory of FinTech. However, we could also see RegTech as a separate phenomenon, evolving through years (see Fig. 2).

In general, the periods Reg1 (1967–2008) and Reg2 (2008–2018) are associated with the digitization of the regulatory authority, while the period Reg3 (2018-present) is related to the formation of an appropriate regulatory framework of the digital age.

More specifically, according to Arner et al. [2], Reg3 is the regulatory term directly linked to the future of RegTech. Concepts such as data dominance and algorithm monitoring now need to be modified as RegTech is used to control how regulations work and who should be subject to those regulations. It's the era marked by the transition from 'Know Your Customer' (KYC) to 'Know Your Data' (KYD). The main obstacle for RegTech is not the technological constraints, but probably the ability of regulators to process and analyze big data.

Therefore, regulators are required to develop systems that allow them to properly monitor and analyze all data. It is certain that the development of regulations has become necessary in order to meet the growing need for cybersecurity.

The technologies with the greatest impact on the FinTech growth are:

*Blockchain technology* Blockchain allows computer systems located in different places to propose, validate transactions and update the files of a common network at the same time and enhance efficiency [44, 55]..

Blockchain information is not stored in a specific location, making malicious attacks more difficult, while the required time for transactions is limited. However, there are also new risks associated with money laundering, inadequate customer protection and tax evasion [20].

A main benefit of Blockchain technology is Smart Contacts which are based on a purely digital and complex computerized protocol and includes complex calculations, multi-party agreements, various forms of encryption and contributes to the make transactions safer. They also improve the ability of conducting contracts and offer easier confirmation that all obligations have been met, while at the same time, the monitoring time is being eliminated and therefore the contractual monitoring costs are reduced [24]. However, there is also a belief that Smart Contracts may create new legal requirements [9].

According to Saripalli [48], Blockchain can facilitate the decentralization of the last mile delivery channel, by enabling peer-to-peer and cashless transactions even among the unbanked population.

*Application Programming Interface* The Application Programming Interface (APIs), is the way of communication for two computer applications in a network, using the same communication code [56]. Through different channels offered by FinTech companies, banks can offer products and services of great flexibility and short time required, thus facilitating innovation [17].

According to Vishnu et al. [53], APIs have the same type of functionality in m-banking. They can be used to authorize the use of banking data by third parties. How banks are evolving over the years is important because, according to the OECD, the activities of the financial sector accounts for between 20 and 30% of the GDP of developed countries.

The open banking model is promoted by the Directive PSD2 and is based on APIs. It is an important data source, as it allows access to system’s data. This adds value to the bank, as through access to multiple data sources it receives valuable, difficult and complicated information. APIs are not a new reality in the banking system, as they are already used in the internal communication between the various infrastructure systems (or nodes). APIs are at the heart of the FinTech revolution, as they influence the way products and services are delivered and used. On the other hand, PSD2 allows access to customer information and communication from authorized third parties. According to Omarini [43], they have been created in order to ensure this compatibility.

*Artificial Intelligence* Artificial Intelligence (AI) is a bigger concept to create intelligent machines that can simulate human thinking capability and behavior, whereas, machine learning is an application or subset of AI that allows machines to learn from data without being programmed explicitly. Its popularity has increased, mainly due to the large volume of digital data, the growing need for data storage and the great progress that has been made in the algorithms that are applied [22].

According to Schlinder et al. [49], the applications of artificial intelligence are diverse and apply, inter alia, to regulatory reporting and data quality, monetary policy and risk analysis, as well as fraud monitoring and detection.

At the central bank level, AI has been integrated into functions that contribute to the identification of microeconomic and macroeconomic indicators [49], supervision, information management and forecasting and detection of malicious activities [22].

The benefits of AI application are manifold. In the financial sector, the use of AI and ML techniques increases efficiency, reduces transaction costs, improves service quality, provides smart investment solutions, increases and boosts customer satisfaction. In addition, AI and ML applications allow the institutions that use them to analyze all customer data to which they have access, learning about their preferences and thus developing specific products and services tailored to customer needs, while improving user experience [22].

Hsu [27] has developed a stock selection model in the S&P 500 and the FTSE 100, applying machine learning methods to enhance the performance of the benchmark for individual investors. The results of this study suggest that machine learning techniques are well applied in stock markets.

Villar and Khan [52], using artificial intelligence procedures, demonstrated how Deutsche Bank successfully automated Adverse Media Screening (AMS), speeding up compliance, increasing coverage for negative media, and drastically reducing false positives.

*Virtual and Augmented Reality* Augmented Reality (AR) and Virtual Reality (VR) are relatively new applications which are based on the principle of interaction. Their cost is still high, as they incorporate desktop, software, headphones, visual content and advertising costs [28]. According to Goldman Sachs, it is estimated that in 2025 the value of the VR market will amount to 25 billion dollars [34]. The technological revolution and the evolving customer base, led financial institutions to introduce AR and VR technology in financial services [28].

*Robo Advisors* Robo Advisors (RA) platforms provide automated portfolio management services that require minimal or even no human intervention, at a significantly lower cost than traditional consultants [47]. Automated service consultants or robotic consultants consist a new challenge in the financial services industry, providing investment, banking and insurance products. A well-known RA is the automated investment advisor in the financial sector [8]. A well-designed RA can be competent, honest and can recommend suitable products to their customers [7]. However, the motivation of those who plan or develop RA may not be objective as the applied algorithms may not be in favor of the customers’ benefit, but in favor of the providers [8]. According to Liu et al. [37], robotics is increasingly being used to automate customer interaction. In addition, robotics improve efficiency and the quality of execution Vishnu et al. [53].

*Cloud Computing* Cloud Computing (CC) is the on-demand availability of computer system resources, especially data storage, data sharing and remotely work through internet access. CC offers flexibility in the provision of services and the saving of resources. However, CC like APIs, if not segregated securely and not adequately monitored may cause serious problems [54].

## Covid-19 pandemic and banking sector challenges

The Covid-19 pandemic has forced countries around the world to accept a new reality as almost everything in people's daily lives has changed dramatically. This new reality has forced financial institutions to move fast to protect both their employees and their customers, changing their operations and serving customers in new ways.

The coronavirus footprint was visible to both consumers and banks with small businesses in their clientele. The steps taken during the pandemic period will shape many banking activities in the future. What is certain, however, is that the need to "Stay Home" is rapidly accelerating the adoption of digital technologies. This increasing use of digital technology means at the same time reduced dependence on traditional banking branches, thus accelerating the transformation of the banking landscape. It is estimated that in China and Italy, just four weeks after the onset of the coronavirus, the increase in consumer digital choices increased by between 10 and 20%. This gained experience by consumers may change their trading behavior in the long run [1].

Currently, the implementation of a dynamic and flexible banking model seems inevitable. During the pandemic, the operation of bank branches was temporarily differentiated by incorporating remote work. If this continues on a more permanent basis, it will obviously affect the number of branches currently in operation as well as the number of bank employees. Such a scenario is expected to accelerate the conversion process of traditional banks to virtual banking, where the customer communicates with a specialized consultant via video call in order to conduct banking transactions, thus developing a model that relies heavily on remote consultants [1]. In the near future, it is very likely that customers will visit the bank branches relatively infrequently, as almost all of the services will be offered through e-banking, mobile banking or virtual banking. Therefore, the challenges are expected to be intense with long-term effects worldwide.

Especially in the case of Greece, the imposition of capital controls in 2015 contributed to the first rapid transformation of banking transactions where the paper currency was replaced by plastic money. The Covid-19 pandemic has pushed this transformation further. As the crisis progresses, we have more and clearer evidence of the impact on the behavior and expectations of customers and businesses but what is certain is that there can be no return to the methods applied before 2019 [1]. Banks need to develop new strategies taking into account certain internal and external factors. The new trends will definitely include great receptivity to digital channels. After the crisis, employees may be more willing to embrace new working models remotely. On the other hand, banks will have to face a prolonged period of low interest rates and reduced profits with tighter balance sheets and higher operating costs due to the new security measures and for their survival they must move immediately with the right decisions.

Thus, we see that although the global financial crisis of 2008 highlighted the seriousness of systemic risks to banks, in the case of the Covid-19 pandemic the risk was entirely due to factors unrelated to the banking system offering banks the opportunity to highlight their role as a systemic stabilizer. Banks need to learn from the two crises that emerged in 2008 and 2019 respectively and proceed directly to their own digital transformation, while at the same time creating a much higher degree of operational and financial resilience [16]. During the recent global crisis, banks were considered to be the biggest problem. Today, however, they are considered to be at the heart of problem solving [11].

## Sample and hypotheses

*Sample* In the context of this study, we conducted questionnaires to collect information from the participants selected in our samples and the hypotheses were tested by non-parametric tests. For the purposes of our study, we use two separate groups and samples, constructing two independent questionnaires, oriented to the needs of our study.

The first sample includes consumers/users of banking products and services, regardless of the banking institution that are customers, who answered a questionnaire structured and tailored to their personal transactional needs, habits and desires. The survey was conducted in Greece during the period 28/12/2019–19/02/2020 where 300 questionnaires were distributed to users of which 241 questionnaires received feedback. 10 questionnaires were removed due to omissions in the answers and we finally obtained 231 valid questionnaires, with an effective rate of 77%. The sample consists of 117 women (50.65%) and 114 men (49.35%). The respondents are mainly under the age of 50 (n = 206, 89.15%) and only 42 (18.18%) of the respondents have not graduated from a higher education institution.

The second pool of our study includes the employees of Greek banks. This survey took place in Greece during the period 10/01/2020 to 12/02/2020. In order to investigate the banks employees’ convictions and opinions related to FinTech, we distributed 148 questionnaires to employees from which we received feedback on 120 questionnaires of which 16 questionnaires were deleted due to omissions. Thus, our final sample consists of 104 valid questionnaires, with an effective rate of 70.3%. The second sample of bank employees consists of 64 women (61.54%) and 40 men (38.46%). Participants under the age of 40 constitute the largest percentage (n = 97, 93.20%) while the 87 employees (83.65%) hold at least an undergraduate university degree.

The sampling for distribution and completion of both users’ and bank employees’ questionnaires followed the rules of sampling. In the case of bank employees, the questionnaires were distributed to employees of both bank branches and headquarters services during the time period mentioned above. Regarding the users, the questionnaires were distributed to citizens outside specified metro central stations during the period 28/12/2019 to 19/02/2020. Both questionnaires are listed in the appendix section at the end of the paper, while the empirical results with the corresponding tables are analyzed at the empirical part of the paper.

A common feature of both our questionnaires is that they include three critical questions to test our hypotheses, regarding the demographic characteristics, gender, age and educational level of the respondents.

*Hypotheses* The users’ questionnaire was constructed by categorizing the questions into four subgroups, which emerged from the research questions and led to the hypotheses testing of the study. A total of twenty questions are included:

- The intention to use FinTech services
- The perception of the usefulness offered by FinTech services
- The trust and the perception of the risk that is integrated in the FinTech services
- The preference between FinTech services and services provided by traditional banking branches

On the other hand, bank employees were asked to answer a questionnaire consisting of thirteen questions structured in four subgroups, according to the research needs and the hypotheses testing:

- The knowledge they have in new technologies
- The degree of adoption of new technologies
- The perceived usefulness of new technologies
- The perception they have regarding the digital transformation and employment

More specifically, the following hypotheses are considered:

- Do users take advantage of the opportunities provided by FinTech companies?
- Do they receive sufficient satisfaction from the use of FinTech services?
- Do they trust FinTech companies and/or banks for securely making transactions?
- Do bank employees have thorough knowledge on new technologies and to what extent they adopt them in their professional environment?
- According to bank employees how useful new technologies are for their work and for customers’ satisfaction and whether there may be a negative impact on future employment?

## Empirical approach

For each variable we examine descriptive statistics average, median, standard deviation and variance and then Kolmogorov Smirnov and Shapiro Wilk tests for normality is applied.

However, as the samples do not follow normal distribution, we apply the non-parametric tests:

- Kruskal–Wallis test.
- Chi-squared (X^2) test for independence.
- rs rank-order Correlation Coefficient (Spearman test).

In our research we are interested to examine which variables from both questionnaires are affected and differentiated by demographic factors, i.e. gender, age and educational level. As the data from both samples do not follow the normal distribution, non-parametric tests at 5% significance level will be tested. When P-value is less than 5%, the examined variable is not independent by each tested demographic factor. Thus, in the next step non -parametric tests follow a single factor (non-parametric dispersion analysis), using Kruskal–Wallis non parametric tests.

We apply the non-parametric Kruskal–Wallis test by ranks, or one-way ANOVA on ranks for testing whether samples originate from the same distribution [18, 35, 50]. This is a way for comparing two or more independent samples of equal or different sizes and tests whether the difference is statistically significant related to their median. It extends the Mann–Whitney U test, which is used for comparing only two groups. The parametric equivalent of the Kruskal–Wallis test is the one-way analysis of variance (ANOVA).

A significant Kruskal–Wallis test indicates that at least one sample stochastically dominates over the other sample. Since it is a non-parametric method, the Kruskal–Wallis test does not assume a normal distribution of the residuals, unlike the analogous one-way analysis of variance. Kruskal–Wallis test is widely used to test small-sized non-parametric models, as it provides exact probabilities for sample sizes even less than about 30 participants. It is indicative that the mechanism behind Kruskal–Wallis test has the possibility to adequately estimate smaller samples, relying on asymptotic approximation for larger sample sizes. Spurrier [51] published exact probability tables for samples as large as 45 participants, while Meyer and Seaman [39, 40] produced exact probability distributions for samples as large as 105 participants. In order to use the Kruskal—Wallis criterion (K-Intependent Samples), we test for the following hypotheses:

The null hypothesis is accepted when P (value) is greater than 0.05, while when P (value) receives price less than 0.05 the null hypothesis is rejected in comparison to the alternative one (H1).

*Validity and Reliability Tests* Before we applied the proposed conceptual model, we have tested the internal reliability and validity of the scales by calculating the Cronbach alpha coefficient. Cronbach's alpha tests the internal consistency among a set of items. It is a scale reliability measure and its value ranges from 0 to 1. When Cronbach alpha value equals to 0 it implies that scale is perfectly unreliable whereas a value 1 suggests that it is perfectly reliable. When Cronbach alpha value is greater than 0.5 the scale is considered reliable and therefore it is acceptable, whereas if it is over 0.7 the scale is considered very reliable. The Cronbach alpha value depends on the number of the items, the inter-item covariance and the average variance.

The reliability of the Cronbach alpha value for the first questionnaire addressed to users is 0.708 whereas, the reliability of the Cronbach alpha value for the second questionnaire addressed to the employees is 0.796.

## Empirical results

The following tables (1 and 2) summarize the cases where we compare the variables and we find different medians (p < 0.05) which implies differences in statistical significance.

For the first, users’ questionnaire, we found the following results, in Table 1:

**Table 1:** Kruskal Wallis test for users

Variables	Gender	Age	Educational level
Knowledge of the existence of digital banks & Fintech companies	p = 0.001		
Knowledge of the existence of digital currencies (cryptocurrencies)	p = 0.000		p = 0.002
Use of Services: TransferWise, Currency Fair, Peer Transfer, Currencies Direct			p = 0.011
Quality of services as a selection factor for a non-banking provider			p = 0.030
Availability of services as a selection factor for a non-banking provider			p = 0.050
Quality significance of services provided			p = 0.020
Availability significance of services provided			p = 0.006
Transactions security as an interesting Fintech area of action		p = 0.007	
Funding as an Interesting Fintech area of action		p = 0.031	
Significance of interaction between customers and banks via mobile		p = 0.001	p = 0.001
Same level of trust for both banks and Fintech firms	p = 0.006		
Risk undertaken (i.e. related to the violation of privatization, phishing etc.) through the use of Fintech		p = 0.039	
Better service provided by Fintech firms than traditional banks		p = 0.039	
Stop using a banking service due to better experience from Fintech		P = 0.037	

On the other hand, from the answers of employees in the second questionnaire we find the results presented in Table 2:

**Table 2:** Kruskal Wallis test for employees

Variables	Gender	Age	Educational level
Evaluation of banks’ innovation	p = 0.011		
Readiness to meet the requirements of new technology			p = 0.010
Facilitation of daily work by using new technologies	p = 0.036		
Effective customer service through technological innovation			p = 0.006
Automation of operations as a threat to traditional banks personnel employment			p = 0.007

*Chi-square Χ*^*2*^* testing* At this stage the Chi-square (Χ^2) test for independence was performed in order to determine whether the questionnaire’s variables are independent, not correlated to the demographic data of the sample. According to the null hypothesis (H0), the variables are independent between each other (p > 0.05) and the alternative hypothesis (H1), the variables are not independent between each other (p < 0.05) but at least some dependence is present. In the following tables (3–7) we present the cases where null hypothesis is rejected (p < 0.05) implying statistical significance for these relationships, meaning that cases on the other hand with evidence of statistical significance imply that variables are not independent.

(A) Empirical analysis of users’ questionnaire

For the users’ questionnaire we can see the following results in Table 3:

**Table 3:** Users’ Χ^2^ independence test according to gender

Variables	P	v
Knowledge of the existence of digital banks & Fintech companies	0.002	12.547
Knowledge of the existence of digital currencies (cryptocurrencies)	0.000	25.218
Recommendation of Fintech services to friends/family	0.041	9.988
Same level of trust for both banks and Fintech firms	0.000	15.658
Better service provided by Fintech firms than traditional banks	0.007	14.196

- Chi-square Χ^*2*^ test for independence according to gender

Based on Table 3 which shows some kind of dependency on gender, we observe that the highest value (v = 25.218) is given to the knowledge of digital currencies, such as Bitcoin or Litecoin. The lowest value (v = 9.988) corresponds to the possibility of recommending FinTech services to friends and the users’ families. Therefore, we can suggest that data with dependency on both genders are mainly evident, indicating the intention for using FinTech services. In addition, there is a statistical significant dependence relationship between trust and gender, as well as a comparison between FinTech companies and traditional banking branches, regardless gender.

- Chi-square Χ^2^ test for independence according to age

In the above Table 4, we can observe some kind of dependency on the users’ age in relation to their view whether FinTech services provide better services relatively to traditional banks as it receives the highest value (v = 26.402). On the contrary, the lowest price (v = 8.896) corresponds to the question that according to users, funding can be consider as an interesting FinTech area of action. Therefore, the questions which show the highest dependence between age and users’ responses include all the variables which examine the trust and the implied risk for FinTech services as well as the questions that compare FinTech with the traditional banks branches. Another dependence emerging relationship is that between users’ age and their intention for using FinTech services.

**Table 4:** Users’ Χ^2^ independence test according to age

Variables	P	v
Knowledge of the existence of digital currencies (cryptocurrencies)	0.005	18.646
Use of Services: CoinBase, Xapo, Armony, airBitz	0.005	18.549
Transactions security as an interesting Fintech area of action	0.007	12.222
Funding as an Interesting FinTech area of action	0.031	8.896
Greater trust in banks for transactions and protection of privatization	0.047	12.742
Same level of trust for both banks and FinTech firms under surveillance	0.043	12.995
Risk undertaken (i.e. related to the violation of privatization, phishing etc.) through the use of FinTech	0.023	23.640
Better service provided by FinTech firms than traditional banks	0.009	26.402
Provision of consulting support services by FinTech companies	0.005	18.676

- Chi-square Χ^2^ test for independence according to educational level

Based on the above Table 5 regarding the questions that have some kind of dependence on users’ education level, the highest value (v = 61.441) corresponds to the importance of interaction between the banks and the use of mobile phones. On the other hand, the question related to the availability of services as a selection factor for non-banking providers corresponds to the lowest value (v = 12.939). So far, the main questions with the highest dependence with the users’ educational level are those which examine the intention to use Fintech services.

**Table 5:** Users’ Χ^2^ independence test according to educational level

Variables	P	V
Main ways of handling banking transactions	0.006	27.521
Knowledge of the existence of digital currencies (cryptocurrencies)	0.007	17.305
Use of Services: TransferWise, Currency, Peer Transfer, Currencies Direct	0.005	18.490
Use of Services: CoinBase, Xapo, Armony, airBitz	0.005	18.446
Quality of services as a selection factor for a non-banking provider	0.003	14.305
Availability of services as a selection factor for a non-banking provider	0.005	12.839
Quality significance of services provided	0.001	33.572
Availability significance of services provided	0.002	30.581
Top choice of FinTech service activity area	0.020	28.194
Significance of interaction between customers and banks via mobile	0.000	61.441
Same level of trust for both banks and FinTech firms under surveillance	0.022	14.794

(B) Empirical analysis of employees’ questionnaire

At this section the analysis focus on the main results derived from the elaboration and test of employees’ questionnaire.

- Chi-square Χ^2^ test for independence according to gender

In the above Table 6, we present only the questions that have some dependence on the gender. The most important results are linked with the existence of dependence between the employees’ gender and the assessment of innovation (ν = 8.821) and the facilitation of daily work with the use of new technologies, such as digitization (ν = 8.894).

**Table 6:** Employees’ Χ^2^ independence test according to gender

Variables	P	V
Evaluation of bank's innovation	0.032	8.821
Facilitation of daily work by using new technologies	0.030	8.924

- Chi-square Χ^2^ test for independence according to educational level

Table 7 shows the employees’ responses being affected by their educational level. These responses are related to the degree of effective customer service through the use of technological innovation (ν = 25.301), the readiness to meet the requirements of new technology (ν = 19.548) and the automation of work as a threat to their employment conditions.

**Table 7:** Employees’ Χ^2^ independence test according to educational level

Variables	P	V
Readiness to meet the requirements of new technology	0.021	19.548
Effective customer service through technological innovation	0.013	25.301
Automation of operations as a threat to banks personnel employment	0.044	17.347

- Chi-square Χ^2^ test for independence according to age

In addition, the Chi-square Χ^2^ test for independence according to the age of the employees does not show any kind of dependence between the respondents’ answers and their age.

Summing up the above analysis, we find that in the first questionnaire addressed to the users, the demographics related to the age and education level are somewhat dependent with several questions from almost every subgroups of the questionnaire, whereas, compared to the gender it seems to be dependent on specific responses related mainly to the knowledge of FinTech services and trust on FinTech services. Concerning the employees, we can come to the conclusion that demographic characteristics of the sample in relation to the gender and education are dependent on specific responses such as the services innovation, work facility, readiness for using FinTech, effectiveness in serving customers and the impact of new technologies for their employment future. On the contrary, dependence is not present in the relationship between the age of the employees and the responses in the questionnaire.

(III) rs rank-order Spearman Correlation Coefficient

Spearman's correlation coefficient or Spearman's r{s}, is a nonparametric measure of ranking correlation as it tests for the existence of statistical dependence between the rankings of two variables. Alternatively, it can assess how well the relationship between two variables is described using a monotonic function. When there are no repeated data values means that a perfect Spearman correlation of + 1 or − 1 occurs as each of the variables is a perfect monotone function of the other. Intuitively, the Spearman correlation between two variables will be high when observations have a similar (or identical for a correlation of 1) ranking between the two variables, and low when observations have a dissimilar (or fully opposed for a correlation of − 1) ranking between the two variables.

According to the null hypothesis (H0) tests show that variables are not related between each other, meaning that it does not seem to have any kind of dependence (p > 0.05). On the other hand, the alternative hypothesis (H1) tests whether variables are correlated to each other, meaning there is a dependence relationship (p < 0.05) between the examined variables. The following Tables 8, 9 show that questionnaire responses reject the null hypothesis (H0) accepting the alternative hypothesis (H1) so there is a dependence relationship.

**Table 8:** Spearman correlation for users

Variables	Demographics	P	r_s_
Knowledge of existence of digital banks and FinTech companies	Gender	0.001	0.225
Knowledge of existence of digital currencies (cryptocurrencies)	Gender	0.000	0.329
	Educational level	0.009	– 0..171
Same level of trust for both banks and FinTech firms	Gender	0.006	0.182
Quality of services as a selection factor for a non-banking provider	Educational level	0.006	– 0..187
Availability of services as a selection factor for a non-banking provider	Educational level	0.007	– 0..185
Use of Services: TransferWise, Currency, Peer Transfer, Currencies Direct	Educational level	0.004	– 0..189
Degree of significance of quality	Educational level	0.026	0.146
Degree of significance of price	Educational level	0.027	0.145
Degree of significance of service availability	Educational level	0.011	0.167
Speed of service as a selection factor for a non-banking provider	Age	0.019	0.160
Transactions security as an interesting FinTech area of action	Age	0.024	0.157
Importance of interaction between customers and banks via mobile	Age	0.000	– 0..238
Greater trust in banks for transactions and protection of privatization	Age	0.002	0.653
Risk undertaken (i.e. related to the violation of privatization, phishing etc.) through the use of FinTech	Age	0.006	0.185

**Table 9:** Spearman correlation for employees

Variables	Demographics	P	r_s_
Evaluation of bank's innovation	Gender	0.010	0.251
Facilitation of daily work by using new technologies	Gender	0.035	0.208
Use of Services: Zopa, Lending Club, Funding Circle, Rate Setter	Age	0.034	– 0..210
Readiness to meet the requirements of new technology	Educational level	0.037	0.208

The above Table 8 illustrates the dependence relationship that exists between the demographic data of the sample and the responses from the users’ questionnaire. Thus, the knowledge of adding digital banks/FinTech companies to the financial sector, the knowledge of digital currencies and the question that examines the equivalence of trust between traditional banks and FinTech companies being under the supervision of the European Supervisory Authorities are positively related to users’ gender. Regarding the respondents’ educational level, the questions related to the degree of price, quality and availability of services importance are positively dependent with users’ educational level. On the other hand, we observe a negative correlation between educational level and the questions related with the knowledge of digital currencies, the choice for specific services such as TransferWise, Currency, Peer Transfer, Currencies Direct, the quality and availability of services as non-selecting a non-banking provider.

In terms of age, there is a positive correlation between the responses the users’ age with and the existence of greater trust of users in traditional banks in terms of transactions and personal data management, increased risk undertaken through the use of FinTech services, the speed as a very important factor of selecting a non-banking provider and the increased transactions insurance as an interesting FinTech area of operating. Last but not least, there is a negative dependence relationship between users’ age and the cooperation with a bank through smart phones.

Table 9 shows the dependence between the demographic characteristics of the employees and the corresponding questionnaire.

Regarding the respondents’ gender, there is a positive dependence between the assessment of innovation of traditional banks and the facilitation of the daily work through the use of new technologies and employees’ gender.

As far as the respondents’ age is concerned, there is a negative dependence between their age and the choice of specific services such as Zopa, Lending Club, Funding Circle and Rate Setter.

In terms of employees’ education, the readiness to meet the requirements of new technology is positively dependent to educational level. Finally, the subgroup which is related to digital transformation and employment is not dependent with any of the sample demographics.

*Main Implications of Empirical Results* Taking into account the empirical surveys conducted at the level of users and bank employees, the main findings that emerged are the following:

- The vast majority of users seem to prefer only financial institutions to conduct their banking transactions. According to the literature, FinTech has great potential and is active in various fields. However, the present study shows that users only know payment services as FinTech's area of activity as almost no other activity was reported in the questionnaire responses. Payment services are the activity of most interest to FinTech companies, while the second most important activity is the trading of shares and investments. It is worth noting that most customers who have used payment services have opted for PayPal. This conclusion is also in line with the answers of the bank employees. This is a clear sign that FinTech services in Greece are still at an early stage of development.
- Although there are several factors that affect the decision to use FinTech services, the security factor seems to be of paramount importance for the users. In the case of trust, the majority of the respondents indicate that they trust traditional banks more than other non-financial institutions. According to the literature, the main advantages of banks are the regulatory compliance and the confidence between the bank and its customers, which is built over the years.
- Regarding the adoption of new technologies by banks, it seems that, admittedly, Greek banks have given the necessary weight to the digital transformation and have made significant investments. The increased pressure on traditional banking institutions to modernize their core business activities is mainly due to the parallel penetration of new technology-oriented companies. From the results of the present study, the digital transformation seems to be a one-way street and will continue to light the way for the technological revolution. Also, the findings of the study show that banks have invested significantly in education, offering employees the opportunity to acquire the necessary skills to meet modern needs and be able to further develop their skills. It seems that Greek banks have largely focused their investment strategy on staff training. Another conclusion is that only a small percentage of bank employees feel fully prepared to face the new technological reality, although based on their educational level, this percentage is expected to be higher soon.
- Regarding their future employment, employees seem to be worried and their prevailing position is that for a job that needs mainly automated movements, there is high risk of dismissal. The majority of respondents believe that artificial intelligence and robotics are likely to replace a wide range of professional skills and pose a risk to their personal work and further professional development. The literature shows that artificial intelligence has a very important role as it intervenes and disrupts the activities in tasks traditionally performed by humans. However, its impact on the work is not clear and opinions vary substantially. Professions that require a high degree of creative intelligence, are less likely to be replaced by smart machines in the next decade.
- Non-parametric tests show that in matters of trust, security and protection of personal data, the majority of the sample shows a preference for traditional banking institutions and seems to be age-dependence. The importance of interaction with the use of smartphones seems to depend on users’ age and educational level. On the other hand, for bank employees it holds that their educational level clearly plays an important role in the degree of readiness and response to new technologies.

## Conclusions

FinTech has come to stay is sure to force reform in many areas, mainly intervening technologically and creating competition. In this way there is a view that argues that business activities that have traditionally been vital to the banking sector are threatened. What is certain is that companies that have decoded developments in time and invested significant capital in technology and human resources will be ready for the digital transformation, which will give them a comparative advantage over other companies.

The best way for banks to stay competitive in the new era, is to implement Open Banking, which is both an opportunity and a threat to banking institutions. The threat comes mainly from the limited ability to control the interaction between banks and their customers. However, the most important element that should not be lost in the new economic landscape is the trust of all stakeholders (banks, FinTech, TechFin, regulators and users). A new open platform between banks and FinTech is sure to be an advanced environment for increasing bank innovation. For example, through the Open Banking environment, customers will be able to view all their accounts and transactions independently of the bank provider on a 24-h basis.

It is obvious that in the coming years we will be able to know who the real winner will be, between the FinTech companies and the banking institutions. According to the existing literature, the one that will prevail will be any more investments to succeed in turning it into a customer-centric organization, even if it is a new coalition or collaboration that will consist of both players [43].

It is accepted that the 4th Industrial Revolution has transformed modern enterprises by causing radical changes in the banking sector. Global banking giants have begun to be challenged by new players, while at the same time FinTech and BigTech are claiming market share, creating major changes in consumer patterns and habits.

The main results of the current study imply that users only know payment services as FinTech's area of activity and prefer to use for their transactions PayPal. It is worth noting that payment services consist the most invested activity for FinTech firms.

Security, trust and protection of privatization seems to be by far the most significant factors affecting users in conducting transactions, using new financial technologies and that is mostly argued by trusting traditional banks more than other non-financial institutions. For their transactions, most of customers use smart phones, whose use depends on users’ age and educational level.

Moreover, banks have invested significantly in education, offering employees the opportunity to acquire the necessary skills to meet modern needs and be able to further develop their skills. However, only a small percentage of bank employees feel fully prepared to face the new technological reality. One equally important conclusion for employees’ part is that they consider themselves in risk of being dismissed due to the automation of many jobs. In addition to the above, for bank employees it holds that their educational level clearly plays an important role in the degree of readiness and response to new technologies.

In conclusion, the present study shows that we must be focused on the new generation that will be the basis for the banking system in the future and those in charge to target the needs and expectations of the "Millennials" while at the same time appropriate investments in technology and knowledge must be planned. Recent literature shows that FinTech companies may look and act like banks, but they are not yet set up as banks. It is also worth mentioning that in cases of pandemics, such as Covid-19, the importance of FinTech is highlighted while financial technology in all areas of activity becomes more important than ever. The coronavirus crisis has helped the banking sector take steps of digital transformation in the short term, something that would otherwise take longer to take place. What is certain is that these new developments will "force" customers to adapt to these unprecedented conditions, while creating new business habits, regardless of age and educational level. Questions such as what is the real relationship between banks and FinTech companies, who is most affected and by whom, are interesting topics for future investigation as well as to repeat the current study after the end of the Covid-19 crisis to compare the results, even expanding the study to incorporate the financial systems of more Member States in the E.U.

The above conclusions must be taken into account by the competent regulatory authorities in order to shield the economy with the necessary institutional framework for the operation of the FinTech companies, as well as the Basel Committee for the reconsideration of Basel regulations.

## Data Availability

The data that support the findings of this study are available from the corresponding author upon reasonable request.
